# Consolidation of the perinatal care system and workload of obstetricians: an ecological study in Japan

**DOI:** 10.3389/fgwh.2023.1030443

**Published:** 2023-04-28

**Authors:** Sanae Hattori, Nobuo Sakata, Miho Ishimaru, Nanako Tamiya

**Affiliations:** ^1^ Department of Health Services Research, Faculty of Medicine, University of Tsukuba, Ibaraki, Japan; ^2^ Health Services Research and Development Center, University of Tsukuba, Ibaraki, Japan; ^3^ Department of Oral Health Promotion, Graduated School of Medical and Dental Science, Tokyo Medical and Dental University, Tokyo, Japan

**Keywords:** perinatal care, delivery, obstetrician, workload, ecological study

## Abstract

**Objectives:**

We examined the relationship between consolidation of delivery and the workload of obstetricians working at perinatal centers.

**Methods:**

We conducted a descriptive analysis using perinatal care areas classified into three types (metropolitan, provincial, and rural). We calculated the Herfindahl–Hirschman Index (HHI) as an index of consolidation and the proportion of the deliveries at clinics as an indicator of the low-risk deliveries and the deliveries per center obstetrician as an indicator of obstetricians’ workload. We used >150 deliveries yearly as an excess indicator. The correlation between the HHI and obstetricians' workload and the proportion of deliveries at clinics was examined using the Pearson correlation coefficient.

**Results:**

The proportion of areas with  >150 deliveries yearly was higher in the consolidated areas. In provincial areas, obstetricians’ workload was positively correlated with the HHI and was negatively correlated with the proportion of deliveries at clinics.

**Conclusions:**

The obstetricians' workload may be increasing where more consolidation occurs. In provincial areas, the center obstetrician's workload could be reduced not only by consolidation but also by sharing the role of handling low-risk deliveries with clinics and hospitals with obstetric units other than perinatal centers.

## Introduction

The shortage of obstetricians is an important and growing global problem (1–4). Many developed countries have consolidated deliveries to perinatal centers to make obstetric care more efficient and to address the shortage of obstetricians (3–8). A 2009 World Health Organization (WHO) report on emergency obstetric care indicated that a comprehensive perinatal center is needed for every 500,000 people (9).

The impact of delivery consolidation on maternal and neonatal health has been previously reported. A population-based study reported that the neonatal mortality rate decreased as the size of maternal units in the delivery facility increased (10). In addition, the proportion of cesarean sections was lower in areas with delivery consolidation than that in those without consolidation (11). Meanwhile, whether all deliveries should be consolidated in perinatal centers regardless of risk remains controversial. Regarding high-risk deliveries, preterm infants had lower mortality and comorbidities when delivered in perinatal centers or institutions with larger delivery units (12, 13). However, regarding low-risk deliveries, medical interventions were more frequent for pregnant women in large hospitals than in small hospitals (14).

Furthermore, no previous studies have shown whether consolidation of deliveries reduces the burden on obstetricians. Only the Ministry of Health Labor and Welfare (MHLW) in Japan report indicates that the burden on obstetricians could be reduced when deliveries and obstetricians were consolidated in perinatal centers (15). However, the number of obstetricians differs between metropolitan and rural areas (16). In areas with few obstetricians, consolidation may increase the burden on center obstetricians. It is necessary to consider whether consolidation should be done by region type.

To the best of our knowledge, no studies have examined the current status of consolidation of the perinatal care system by region type and the impact of consolidation on the workload of obstetricians. Therefore, by using Japan's national survey data, we conducted a descriptive analysis of consolidation of delivery in perinatal centers by region type and examined the relationship between consolidation and the number of deliveries per obstetrician.

## Methods

### Design and data source

This was a cross-sectional ecological study using data from the Hospital Bed Function Report (17) and the Medical Facility Survey in 2017 in Japan (18). The Hospital Bed Function Report is a survey that medical facilities with beds are obliged to report yearly, compiled into a database by the MHLW. It includes >140 items related to the medical functions possessed by each medical institution, specific facilities, medical staffing, and the medical care content. The Medical Facility Survey is a survey that all medical facilities must report every year. This study aimed to identify trends in the number of medical facilities, beds, and medical specialties in each region, containing the numbers of physicians in each department. Both reports contain the name of the individual hospital.

Medical institutions with at least one delivery per year were selected for this study. Institutions were classified into three types: perinatal centers (19), general hospitals, and clinics with <20 beds. In Japan, >99% of deliveries occur at medical institutions (20), and deliveries at midwifery units, homes, or other sites were not included.

### Categorization of regions

We examined the regional characteristics of the perinatal care area (21). Perinatal care areas are established at the prefectural level to improve regional perinatal care based on geographical characteristics, the number of births, and the number and size of medical facilities.

To describe the characteristics of perinatal care areas, we categorized the areas into three types: metropolitan, provincial, and rural. We defined the area types using the following criteria according to the previous study (22): (1) metropolitan areas (population density ≥1,000 people/km^2^ and population ≥1,000,000); (2) provincial area (population density of 200–1,000 people/km^2^ and population ≥300,000); and (3) rural areas (population density <200 people/km^2^ and population <300,000).

### Indicator of obstetricians' workload

The indicator of the workload of center obstetricians in this study was defined as the number of deliveries per center obstetrician (abbreviated as delivery number/center obstetrician). Henceforth, obstetricians/gynecologists who work in a perinatal care center are referred to as “center obstetricians”. We calculated this indicator by dividing the number of deliveries at perinatal centers in a perinatal care area by the number of center obstetricians in that area. Furthermore, we used >150 deliveries annually as an excess indicator. According to the previous study, this indicator is considered a proxy for the burden of center obstetricians, which demonstrated to compare the burden of obstetricians in different medical regions (23). The Japanese Society of Obstetrics and Gynecology reported that the number of deliveries one obstetrician working at a hospital could handle without difficulty is limited to 150 cases yearly. The percentage of medical regions that exceed that standard was also calculated.

### Indicators for consolidation of delivery by regional

We used (1) the Herfindahl–Hirschman Index (HHI) and (2) the proportion of deliveries at clinics as indicators for regional consolidation of delivery.

(1) The term “HHI” is a commonly used indicator of market concentration (24). The HHI has also been used as an indicator of consolidation in medicine. In a previous study, the HHI was used as a measure of interhospital competition to investigate the relationship between interhospital competition and hospital charges or costs in neurosurgery (25). The HHI for a given market equals the sum of squared shares for each hospital in the market. For example, for a market consisting of four hospitals with shares of 40%, 30%, 20%, and 10%, the HHI is 0.3 (0.16 + 0.09 + 0.04 + 0.01 = 0.3). HHI ranges continuously from 0 (significant competition) to 1 (monopoly). Markets with an HHI between 0.15 and 0.25 are considered moderately consolidated, while markets with an HHI >0.25 are considered highly consolidated (26).

We calculated the HHI in this study as follows:

$$\mathrm{HHI}=\;\sum {\left(\frac{\mathrm{number}\;\mathrm{of}\;\mathrm{deliveries}\;\mathrm{at}\;\mathrm{each}\;\mathrm{medical}\;\mathrm{institution}}{\mathrm{number}\;\mathrm{of}\;\mathrm{deliveries}\;\mathrm{in}\;\mathrm{perinatal}\;\mathrm{care}\;\mathrm{area}}\right)}^{2}$$

(2) There is a debate as to whether low-risk deliveries should also be consolidated. Therefore, low-risk deliveries consolidated in centers should be explored. Furthermore, clinics have fewer medical equipment than hospitals. Since clinics are primarily places that handle low-risk deliveries, the proportion of deliveries at clinics as an indicator of low-risk deliveries was used. This proportion was calculated by dividing the number of deliveries at clinics in the perinatal care area by the total number of deliveries in that area.

### Statistical analyses

First, we analyzed the number of birthing facilities, deliveries, and obstetricians in each perinatal care area by region. Then, we used Pearson's correlation coefficient (*r*) to examine the workload of center obstetricians as a result of consolidated delivery: the correlation between HHI and the delivery number/center obstetrician was calculated. We also examined the correlation between the HHI and the delivery number/center obstetrician when deconsolidated: the correlation between the percentage of clinic deliveries and the delivery number/center obstetrician was calculated. A *P*-value <0.05 indicated a significant difference. All analyses were performed using EZR version 1.53 (The R Foundation for Statistical Computing, Vienna, Austria), an R graphical user interface (27).

## Results

The overall number of perinatal care areas was 284 in Japan. Table 1 shows the categorized perinatal care areas. There were 43 metropolitan areas (15.1%), 144 provincial areas (50.7%), and 97 rural areas (34.2%). The number of perinatal medical regions with designated perinatal care centers was 40 (93.0%) in metropolitan areas, 116 (80.6%) in provincial areas, and 44 (45.4%) in rural areas.

**Table 1:** Characteristics of each perinatal care area after categorization.

Characteristics	Metropolitan area	Provincial area	Rural area
Perinatal care areas, *n*	43	144	97
Total population (IQR)	1,225,772 (841,003–1,551,816)	347,895 (227,300–531,132)	86,884 (57,255–130,585)
Total delivery facilities, mean (SD)	19.3 (10.8)	7.6 (4.7)	1.9 (1.3)
Perinatal centers, mean (SD)	3.4 (2.5)	1.5 (1.1)	0.5 (0.7)
General hospitals, mean (SD)	6.1 (3.9)	1.9 (1.6)	0.7 (0.7)
Clinics with beds, mean (SD)	9.8 (4.2)	4.2 (3.1)	0.7 (0.9)
Deliveries per month, mean (SD)	768.5 (488.9)	247.3 (169.1)	44.0 (35.1)
Number of obstetricians, mean (SD)	94.9 (58.1)	28.6 (22.0)	5.0 (3.3)
HHI, median (IQR)	0.085 (0.064–0.130)	0.196 (0.147–0.300)	0.572 (0.500–1.000)
Proportion of deliveries in clinics with beds, % (SD)	42.0 (18.6)	49.3 (24.7)	28.8 (33.3)
Perinatal care areas with a center, *n* (%)	40 (93.0)	116 (80.6)	44 (45.4)
Areas with no delivery facilities, *n* (%)	0 (0.0)	0 (0.0)	8 (8.2)

Values represent averages for perinatal care areas (total population value represents the median).

SD, standard deviation; IQR, interquartile range.

Table 2 shows the characteristics of each perinatal care area with perinatal centers (excluding medical regions without a perinatal center). The mean number (standard deviation, SD) of center obstetricians was 98.7 (57.7) in metropolitan areas, 33.0 (22.2) in provincial areas, and 6.9 (3.2) in rural areas. The delivery number/center obstetrician was 7.3 (6.7) in metropolitan areas, 9.1 (5.8) in provincial areas, and 13.1 (8.2) in rural areas. The median HHI was 0.083 [interquartile range (IQR), 0.064–0.123] for metropolitan areas, 0.178 (0.132–0.256) for provincial areas, and 0.518 (0.394–1.000) for rural areas. The proportion of perinatal care areas where the delivery number/center obstetrician exceeds 150 yearly is 12.5% in metropolitan areas, 25.9% in provincial areas, and 40.9% in rural areas.

**Table 2:** Characteristics of the average of each perinatal care area in the designated perinatal centers (excluding medical regions without a perinatal center).

Characteristics	Metropolitan area (*N* = 40)	Provincial area (*N* = 116)	Rural area (*N* = 44)
Total obstetricians, mean (SD)	98.7 (57.7)	33.0 (22.2)	6.9 (3.2)
Center obstetricians, mean (SD)	36.5 (28.1)	12.6 (11.6)	3.2 (1.7)
Deliveries per obstetrician (all facilities), mean (SD)	8.5 (2.3)	9.4 (2.9)	9.3 (3.8)
Deliveries per center obstetrician, mean (SD)	7.3 (6.7)	9.1 (5.8)	13.1 (8.2)
HHI, median (IQR)	0.083 (0.064–0.123)	0.178 (0.132–0.256)	0.518 (0.394–1.000)
Proportion of deliveries in clinics with beds, % (SD)	41.3 (18.9)	44.6 (21.9)	24.5 (28.3)
Perinatal care areas where the number of deliveries per center obstetrician exceeds 150 per year, *n* (%)	5 (12.5)	30 (25.9)	18 (40.9)

Values represent averages for perinatal care areas. HHI value represents the median.

HHI, Herfindahl–Hirschman Index; SD, standard deviation; IQR, interquartile range.

**Figure 1:**
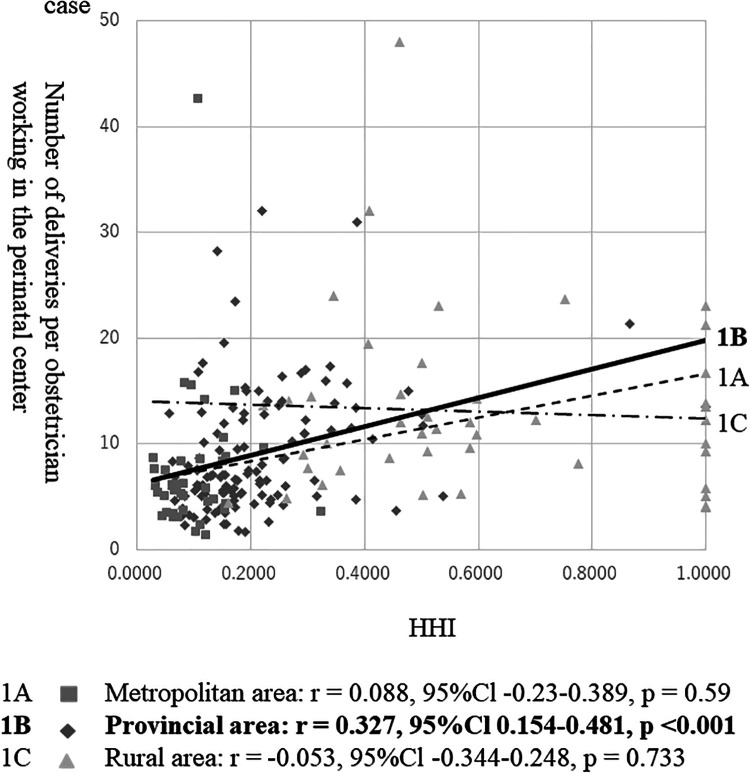
Correlation between HHI and the number of deliveries per obstetrician working in the perinatal center. CI, confidence interval. Note: The analysis excludes perinatal care areas without perinatal centers.

Figure 1 shows the correlation between HHI and the delivery number/center obstetrician. The delivery number/center obstetrician was positively correlated with the HHI in provincial areas (*r* = 0.327, *P* < 0.001). However, the correlations were not significant in metropolitan (*r* = 0.088, *P* = 0.590) or rural areas (*r* = −0.053, *P* = 0.733).

**Figure 2:**
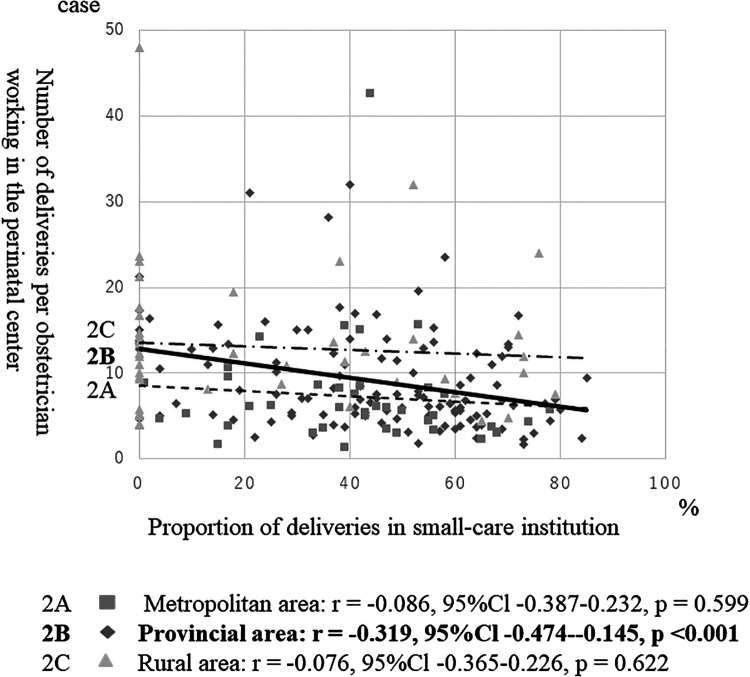
Correlation between the proportion of deliveries in clinics with beds and the number of deliveries per obstetrician working in the perinatal center. CI, confidence interval. Note: The analysis excludes perinatal care areas without perinatal centers.

Figure 2 shows the correlation between the proportion of deliveries at the clinic and the delivery number/center obstetrician. The delivery number/center obstetrician was negatively correlated with the proportion of deliveries at clinics in provincial areas (*r* = −0.319, *P* < 0.001). The correlations were not significant in metropolitan (*r* = −0.086, *P* = 0.599) or rural (*r* = −0.076, *P* = 0.622) areas.

## Discussion

This study is the first to evaluate the consolidation of perinatal care by regional type. We then found that the proportion of areas with >150 delivery numbers/center obstetricians yearly was higher in the consolidated areas. Consolidation was little occurring in the metropolitan areas but was occurring in the other areas.

The metropolitan areas had more delivery facilities and few consolidations. The present study showed that the mean delivery number/center obstetrician in metropolitan areas was relatively lower than that in provincial or rural areas because a large number of obstetricians work there. Previous studies have also shown that consolidation in metropolitan areas is unnecessary (28). The present results are consistent with these findings. Moreover, a regional analysis report revealed no difference in obstetric outcomes between small and large hospitals, and authors concluded that safety alone is not a reason to proceed with consolidation (6). Therefore, we believe that consolidation may not be necessary in urban areas where there are many obstetricians and medical resources.

In the provincial areas, our study found a weak positive correlation between the consolidation of delivery and the delivery number/center obstetrician. This may indicate that delivery consolidation is increasing the burden on center obstetricians. This is because the number of obstetricians is lower than in metropolitan areas. However, we found a weak negative correlation between the proportion of deliveries at clinics and the delivery number/center obstetrician. This may indicate that clinics are responsible for low-risk deliveries, which may reduce the burden on center obstetricians. Previous studies have reported that the incidence of perinatal comorbidities in low-risk pregnant women did not differ depending on delivery facility types if referral systems were well established (6, 7, 29–31). Such a division of delivery roles (increasing the percentage of deliveries at clinics) may be effective in areas where obstetricians are in short supply.

There were fewer delivery facilities in rural areas, and most medical areas had an HHI of 1. Consolidation was inevitable because there were few obstetricians. Therefore, this could not be adequately assessed in this study. Further, prior studies have reported that unplanned birth outside an institution is associated with long travel time to delivery facilities and higher perinatal mortality rates. (32). A previous study from France also reported that the closure of delivery facilities has negatively affected pregnant women in rural areas due to increased travel time (33). Thus, the problem in the rural areas is areas is the lack of appropriate access to delivery facilities rather than consolidation.

Our study showed that 25.9% of provincial and 40.9% of rural areas had >150 deliveries per center obstetrician among perinatal care areas with the perinatal center. We found that obstetrician workload varied by region. One way to improve the working environment for obstetricians is to plan the allocation of obstetricians from metropolitan areas, where there are many obstetricians, to rural areas. Globally, the shortage of physicians is serious in rural areas, and it is difficult to attract physicians to rural areas. Nevertheless, to address this issue, some countries are working on training programs in rural medicine for medical students and young doctors (34–38). Different studies have reported that the programs which provide a rural-oriented medical curriculum and clinical training in rural areas are effective in training physicians who wish to practice in rural areas after graduation. Considering these reforms, it is expected that the number of physicians working in rural areas will increase. Another way is to limit patients delivering in perinatal centers to medium- to high-risk pregnant women. For example, midwifery facilities in the Netherlands provide primary perinatal care for low-risk pregnant women, and pregnant women at middle to high risk categories are referred to a delivery facility with an obstetrician (7). It may be better to share deliveries at existing facilities rather than consolidate to reduce the workload in provincial or rural areas where obstetricians are in short supply. However, previous literatures supporting consolidation pointed out that higher neonatal mortality rate in municipalities with a large proportion of deliveries performed in small units might be attributed to the inadequacy of identifying high risk deliveries (8, 39). As a premise for the division of roles, significant efforts must be invested to identify possible risk factors before delivery.

The present study has several limitations. First, the delivery number/center obstetrician was used as a measure of the burden on the center obstetrician. However, the measure did not reflect the working environment of obstetricians, such as working hours, the number of night shifts, or subjective evaluations. Therefore, this is not a complete indication of the burden on obstetricians working at the center. Further research should be conducted by collecting individual-level workload data to investigate workload among center obstetricians. Second, the data used in this study did not distinguish between the types of delivery, such as vaginal delivery, anesthesia-controlled delivery, and scheduled or emergency cesarean section. Therefore, the number of the delivery is also not a complete indication of the burden on the center obstetrician. Third, some perinatal centers have midwifery units (40). Thus, our data could not distinguish between midwifery-led deliveries and deliveries wherein obstetricians intervened. Therefore, the delivery number/center obstetrician in the center may be overestimated in some areas.

In conclusion, we found that consolidation varied by region. The burden on obstetricians may be increasing in places where consolidation is taking place. We considered that in the provincial areas, the center obstetrician's workload could be reduced not only by consolidation but also by sharing the role of handling low-risk deliveries with clinics and hospitals with obstetric units other than perinatal centers. Further studies may be needed to assess obstetrician workloads in more detail.

## Data Availability

The original contributions presented in the study are included in the article/Supplementary Material; further inquiries can be directed to the corresponding authors.

## References

[1] Anderson BL Hale RW Salsberg E Schulkin J. Outlook for the future of the obstetrician–gynecologist workforce. Am J Obstet Gynecol. (2008) 199(88):e1–8. 10.1016/j.ajog.2008.03.013 18456228

[2] Loy CS Warton RB Dunbar JA. Workforce trends in specialist and GP obstetric practice in Victoria. Med J Aust. (2007) 186:26–30. 10.5694/j.1326-5377.2007.tb00784.x 17229030

[3] Pilkington H Blondel B Carayol M Breart G Zeitlin J. Impact of maternity unit closures on access to obstetrical care: the French experience between 1998 and 2003. Soc Sci Med. (2008) 67:1521–9. 10.1016/j.socscimed.2008.07.021 18757128

[4] Noriko E. Policies to secure obstetricians: a survey in fifteen countries. *Working Paper in Japan Medical Association Research Institute*. (2009) p. 185 (in Japanese).

[5] Bradow J Smith SD Davis D Atchan M. A systematic integrative review examining the impact of Australian rural and remote maternity unit closures. Midwifery. (2021) 103:103094. 10.1016/j.midw.2021.103094 34329966

[6] Hemminki E Heino A Gissler M. Should births be centralized in higher level hospital? Experiences from regionalised health care in Finland. BJOG. (2011) 118:1186–95. 10.1111/j.1471-0528.2011.02977.x 21609379

[7] van den Berg LMM Gordon BBM Kleefstra SM Martijn L van Dillen J Verhoeven CJ Centralisation of acute obstetric care in the Netherlands: a qualitative study to explore the experiences of stakeholders with adaptations in organisation of care. BMC Health Serv Res. (2021) 21:1233. 10.1186/s12913-021-07269-4 34774037 PMC8590329

[8] Moster D Lie RT Markestad T. Neonatal mortality rates in communities with small maternity units compared with those having larger maternity units. BJOG. (2001) 108:904–9. 10.1111/j.1471-0528.2001.00207.x 11563458

[9] WHO. Monitoring emergency obstetric care: a handbook (2009). Available at: https://apps.who.int/iris/handle/10665/44121 (Accessed February 1, 2021).

[10] Moster D Lie RT Markestad T. Relation between size of delivery unit and neonatal death in low risk deliveries: population based study. Arch Dis Child Fetal Neonatal Ed. (1999) 80:F221–5. 10.1136/fn.80.3.f221 10212086 PMC1720939

[11] Maeda E Ishihara O Tomio J Sato A Terada Y Kobayashi Y Cesarean section rates and local resources for perinatal care in Japan: a nationwide ecological study using the national database of health insurance claims. J Obstet Gynaecol Res. (2018) 44:208–16. 10.1111/jog.13518 29094429

[12] Binder S Hill K Meinzen-Derr J Greenberg JM Narendran V. Increasing VLBW deliveries at subspecialty perinatal centers via perinatal outreach. Pediatrics. (2011) 127:487–93. 10.1542/peds.2010-1064 21321032 PMC4172030

[13] Sasaki Y Ishikawa K Yokoi A Ikeda T Sengoku K Kusuda S Short- and long-term outcomes of extremely preterm infants in Japan according to outborn/inborn birth status. Pediatr Crit Care Med. (2019) 20:963–9. 10.1097/PCC.0000000000002037 31232855 PMC6784765

[14] Engjom HM Morken NH Norheim OF Klungsøyr K. Availability and access in modern obstetric care: a retrospective population-based study. BJOG. (2014) 121:290–9. 10.1111/1471-0528.12510 24283373 PMC4253080

[15] Ministry of Health LaW. Guidelines for the establishment of a perinatal care system (2020). Available at: https://www.mhlw.go.jp/content/10800000/000662977.pdf (Accessed February 1, 2021).

[16] Akatsuki K Masao Y Yasuko S Koji T Nobuo T Yoshiko U Trends of geographic and age distribution of obstetrician-gynecologists and pediatricians with contrast to the decrease in the number of children in Japan. Jpn J Health Human Ecol. (2001) 67:291–304. 10.3861/jshhe.67.291

[17] Hospital Bed Function Report (2017). Available at: https://www.mhlw.go.jp/stf/seisakunitsuite/bunya/open_data_00002.html (Accessed January 15, 2021).

[18] Medical Facility Survey (2017). Available at: https://www.mhlw.go.jp/toukei/saikin/hw/iryosd/17/ (Accessed January 15, 2021).

[19] Ministry of Health, Labour and Welfare. List of perinatal care centers. Available at: https://www.mhlw.go.jp/stf/seisakunitsuite/bunya/0000186912.html (Accessed January 15, 2021).

[20] Ministry of Health, Labour and Welfare. Demongraphic survey (2017). Available at: https://www.mhlw.go.jp/toukei/list/79-1.html (Accessed January 15, 2021).

[21] Ministry of Health, Labour and Welfare. Perinatal care areas. Available at: https://www.mhlw.go.jp/stf/seisakunitsuite/bunya/0000186912.html (Accessed January 15, 2021).

[22] Masatoshi Ishikawa TT. Quantitative analysis of Japanese medical service level of each medical region. J Jpn Assoc Health Care Adm. (2013) 7:75–82. 10.11202/jaha.7.75 (in Japanese).

[23] Matsumura K. The analysis of the obstetrics supply system with the secondary medical region database and the problems. J Jpn Assoc Health Care Adm. (2013) 7:107–19. 10.11202/jaha.7.107 (in Japanese).

[24] Akomea YS Adusei M. Bank recapitalization and market concentration in Ghana’s banking industry: a Herfindahl–Hirschman Index analysis. Glob J Bus Res. (2013) 7:31–45. Available at SSRN: https://ssrn.com/abstract=2148565.

[25] Tang OY Rivera Perla KM Lim RK Yoon JS Weil RJ Toms SA. Interhospital competition and hospital charges and costs for patients undergoing cranial neurosurgery. J Neurosurg. (2020) 135:1–12. 10.3171/2020.6.jns20732 33007751

[26] Herfindahl–Hirschman Index. US Department of Justice. Available at: https://www.justice.gov/atr/herfindahl-hirschman-index (Accessed May 9, 2022).

[27] Kanda Y. Investigation of the freely available easy-to-use software “EZR” for medical statistics. Bone Marrow Transplant. (2013) 48:452–8. 10.1038/bmt.2012.244 23208313 PMC3590441

[28] Jordan H Roderick P Martin D Barnett S. Distance, rurality and the need for care: access to health services in south west England. Int J Health Geogr. (2004) 3:21. 10.1186/1476-072X-3-21 15456514 PMC524184

[29] Birthplace in England Collaborative Group; Brocklehurst P Hardy P Hollowell J Linsell L Macfarlane A McCourt C. Perinatal and maternal outcomes by planned place of birth for healthy women with low risk pregnancies: the birthplace in England national prospective cohort study. Br Med J. (2011) 343:d7400. 10.1136/bmj.d7400 22117057 PMC3223531

[30] Lotshaw RR Phillippi JC Buxton M McNeill-Simaan E Newton JM. A collaborative model of a community birth center and a tertiary care medical center. Obstet Gynecol. (2020) 135:696–702. 10.1097/AOG.0000000000003723 32028505

[31] Monk A Tracy M Foureur M Grigg C Tracy S. Evaluating midwifery units (EMU): a prospective cohort study of freestanding midwifery units in New South Wales, Australia. BMJ Open. (2014) 4:e006252. 10.1136/bmjopen-2014-006252 25361840 PMC4216868

[32] Engjom HM Morken NH Hoydahl E Increased risk of peripartum perinatal mortality in unplanned births outside an institution: a retrospective population-based study. AJOG. (2017) 217(2):210.e1–210.e12. 10.1016/j.ajog.2017.03.033 28390672

[33] Combier E Charreire H Le Vaillant M Michaut F Ferdynus C Amat-Roze JM Perinatal health inequalities and accessibility of maternity services in a rural French region: closing maternity units in Burgundy. Health & Place. (2013) 24:225–33. 10.1016/j.healthplace.2013.09.006 24177417

[34] Roberts A Foster RO Dennis M Davis L Wells J Bodemuller MF An approach to training and retaining primary care physicians in rural Appalachia. Acad Med. (1993) 68(2):122–5. 10.1097/00001888-199302000-00003 8431228

[35] Strasser R Neusy AJ. Context counts: training health workers in and for rural and remote areas. Bull World Health Organ. (2010) 88(10):777–82. 10.2471/BLT.09.072462 20931063 PMC2947041

[36] Geyman J Hart G Norris T Coombs J Lishner D. Educating generalist physicians for rural practice: how are we doing? J Rural Health. (2000) 16(1):56–80. 10.1111/j.1748-0361.2000.tb00436.x 10916315

[37] Holst J Normann O Herrmann M. Strengthening training in rural practice in Germany: new approach for undergraduate medical curriculum towards sustaining rural health care. Rural Remote Health. (2015) 15(4):3563. 26564099

[38] Sullivan MH Denslow S Lorenz K Dixon S Kelly E Foley KA. Exploration of the effects of rural obstetric unit closures on birth outcomes in North Carolina. J Rural Health. (2020) 37(2):373–84. 10.1111/jrh.12546 33289170

[39] Heller G Richardson DK Schnell R Misselwitz B Künzel W Schmidt S. Early-neonatal deaths in low-risk births by the size of delivery units in Hesse, Germany 1990–1999. Int J Epidemiol. (2002) 31(5):1061–8. 10.1093/ije/31.5.1061 12435785

[40] Bradford L Glaser G. Addressing physician burnout and ensuring high-quality care of the physician workforce. Obstet Gynecol. (2021) 137:3–11. 10.1097/AOG.0000000000004197 33278277

